# Stochastic Models of Lymphocyte Proliferation and Death

**DOI:** 10.1371/journal.pone.0012775

**Published:** 2010-09-30

**Authors:** Anton Zilman, Vitaly V. Ganusov, Alan S. Perelson

**Affiliations:** 1 Theoretical Biology and Biophysics Group, Theoretical Division, Los Alamos National Laboratory, Los Alamos, New Mexico, United States of America; 2 Center for Nonlinear Studies, Theoretical Division, Los Alamos National Laboratory, Los Alamos, New Mexico, United States of America; 3 Department of Microbiology, University of Tennessee, Knoxville, Tennessee, United States of America; Albert Einstein College of Medicine, United States of America

## Abstract

Quantitative understanding of the kinetics of lymphocyte proliferation and death upon activation with an antigen is crucial for elucidating factors determining the magnitude, duration and efficiency of the immune response. Recent advances in quantitative experimental techniques, in particular intracellular labeling and multi-channel flow cytometry, allow one to measure the population structure of proliferating and dying lymphocytes for several generations with high precision. These new experimental techniques require novel quantitative methods of analysis. We review several recent mathematical approaches used to describe and analyze cell proliferation data. Using a rigorous mathematical framework, we show that two commonly used models that are based on the theories of age-structured cell populations and of branching processes, are mathematically identical. We provide several simple analytical solutions for a model in which the distribution of inter-division times follows a gamma distribution and show that this model can fit both simulated and experimental data. We also show that the estimates of some critical kinetic parameters, such as the average inter-division time, obtained by fitting models to data may depend on the assumed distribution of inter-division times, highlighting the challenges in quantitative understanding of cell kinetics.

## Introduction

After activation by encounter with an antigen, B and T lymphocytes start to proliferate and rapidly expand their numbers. This expansion phase is followed by a period of population contraction resulting in only a small fraction of the expanded population surviving and entering the memory cell pool [1]–[3]. The kinetics of the expansion and contraction affect the speed of antigen clearance and the clinical course of disease [1]. At present, only some of the factors that regulate the differentiation, expansion and contraction of populations of activated B and T cells are known, and the picture of the dynamics of their proliferation and death is incomplete [1], [2], [4]–[10]. In order to dissect the contribution of various factors involved in regulation of lymphocyte kinetics, such as the precursor cell frequency, cell division rate, the availability of antigen, the rate of its presentation, and its affinity to the T cell receptor [2], [4], [11]–[14], a quantitative approach is necessary [11], [15]. Precise estimation of division and death rates during different phases of the immune response is important as it provides input for analyses of intra- and extra-cellular mechanisms giving rise to the observed population behavior.

A large experimental effort has been devoted to uncovering the detailed kinetics of the expansion and contraction of T cells during the response to intracellular pathogens [1], [2], [7]–[11]. Labeling cells using carboxyfluorescein succinimidyl ester (CFSE), an intracellular fluorescent dye that dilutes approximately two-fold as a cell divides, combined with advances in flow cytometry techniques, allows one to quantitatively follow the proliferation and death of large numbers of cells over 6–8 divisions [11], [16], [17]. Interpreting the results from CFSE labeling experiments poses a number of conceptual and methodological challenges. In particular, they necessitate development of both models and computational tools for extracting parameters that characterize the rates of cell activation, proliferation and death. It is also crucial to have a theory that incorporates the distribution of inter-division and death times and the generation structure of the dividing and dying lymphocytes, as well as the effects of variation and noise in the dynamics of lymphocyte populations [15], [18]–[24]. Taking into account the full shape of the inter-division time distribution function is important even for such a relatively simple question as determination of the mean number of cells as a function of time [25].

Several approaches have been proposed recently to provide a quantitative description of the dynamics of populations of proliferating and dying lymphocytes, and to analyze experimental data.

One approach is based on the use of ordinary differential equation (ODE) models. The simplest of these models track the total population of responding lymphocytes. The population may be split into sub-populations, such as resting, activated and memory cells. These models are useful due to their computational convenience and they provide a means for estimation of average birth and death rates in the population [26]–[28].

Extensions of the ODE models can explicitly take into account the generation structure and the variation in the inter-division and death times of lymphocytes. Here, for example, one can write equations for the number of lymphocytes that have divided 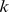 times, 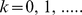, with proliferation and death rates that depend on the “division class” 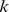 [19], [24], [29], [30]. This class of models is convenient, as it admits analytical treatment and in many cases provides a good qualitative description of the dynamics of the populations of proliferating and dying lymphocytes. However, such models, which typically involve systems of linear differential equations, implicitly assume that the probability distributions of the inter-division and death times are exponential. However, the exponential distribution overestimates the probability that a cell divides shortly after the previous division. In reality, cells are unlikely to divide (or die) immediately after the previous division [23]. Due to this, such models do not provide an adequate quantitative description of lymphocyte proliferation and death, and in many cases cannot be used for quantitative extraction of kinetic parameters [24].

Such models can be extended to include more realistic inter-division time distributions using the Smith-Martin model of the cell cycle [18]–[20], [24], [30]–[34]. In the Smith-Martin model, the cell cycle is divided into two phases: an 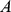 state, whose length is exponentially distributed, and a 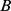 phase of fixed length. In the 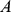 state, the cell grows and accumulates mass. After a certain checkpoint in the cell cycle is passed, the cell becomes committed to division, and enters the 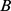 phase, where DNA is replicated and the cell cycle is completed ending with the birth of two new cells. Both progeny are born into the 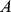 state, and the cycle starts anew. The Smith-Martin model provides a more realistic approximation for lymphocyte inter-division times than models that assume an exponential distribution of inter-division times, such as the ODE models. As a result, Smith-Martin type models provide a quantitatively better description of the dynamics of the populations of proliferating and dying lymphocytes, and of their generation structure, especially when the rates of cell proliferation are high [18], [20], [24], [32].

Recently, another class of models, based on general probability theory has been introduced. These theories incorporate arbitrary (or experimentally motivated) inter-division and death time distributions [15], [22], [23], [35]–[37]. Assuming that the death and birth processes inside each cell are independent, Leon et al. [37] and Hodgkin and coworkers [23], [35], [36] developed a framework that allows one to predict mean numbers of cells in different division classes for arbitrary, generation-dependent distributions of birth and death times. This approach has been extended using the theory of branching processes to include variation in the number of cells per division due to stochasticity in cell division and death [36], [38], [39].

Finally, age-structured population models of the McKendrick–von Foerster type [40], [41] have been used for analysis of lymphocyte population kinetics [20], [36], [42].

All these models are similar in the sense that they describe the potentially complicated underlying biological processes of cellular proliferation and death in terms of effective birth and death parameters. However, at the first glance, the aforementioned models are substantially different in some aspects. For instance, the ODE based models describe cell division in terms of a continuous process characterized by a single birth rate. By contrast, models based on probability theory and branching processes [23], [36]–[39] represent cell division as a discrete event. Finally, Smith-Martin type models [18], [24], [32] make specific assumptions about the progression of the cell cycle. While all these types of models have been used to describe lymphocyte proliferation and death and to estimate the birth and death rates, it still remains unclear to what extent the models are inter-changeable and to what extent the estimates of parameters depend on the choice of a specific model and on the choice of the inter-division and death distributions.

In this paper, we compare different models and their applicability to estimation of parameters from experimental data. Based on and extending previous work, we develop a general quantitative framework, which rigorously derives existing models, elucidates connections between them, and allows us to examine the underlying approximations involved in these models. The framework provides a computationally simple tool for analysis of the dynamics of expanding and contracting lymphocyte populations, complementary to the existing methods. The framework is based on the theory of branching processes [41], [43] and age-structured populations models [40].

The paper is organized as follows. We first develop a theory of the generation structure of the population of dividing and dying cells, based on the theory of age-structured populations and derive expressions for the number of cells that have undergone a given number of divisions. We then use the theory of branching processes to show that this alternative approach gives the same predictions regarding the numbers of cells in a particular division class as the theory of age-structured populations. Next, we show that the models commonly used in the literature all can be derived within the unified framework presented in this paper. We then explore the plausibility of our modeling approach to the estimation of rates of cell division and death using simulated and experimental data.

## Results

### Age-structured Populations

In this section, we use the theory of age-structured populations to compute the number of cells that have undergone a given number of divisions, for an arbitrary distribution of inter-division and death times. Apart from mathematical rigor, the age-structured formulation has the advantage of being easily generalizable to more complicated situations, such as inclusion of time-dependent birth and death rates that might arise from cytokine regulation, dependence of cell properties on the cell age, asymmetric divisions, etc.

Knowing the distribution of inter-division times is critical for determining from experimental measurements the parameters commonly used to describe the kinetics of the immune response, such as the average inter-division time. To exemplify this point, consider a population of cells that, starting with a single individual at time 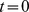, expand with an average *measured* rate 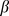, so that the total number of cells at time 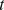 is 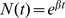 and the population doubling time is 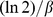. For example, for a population of CD8+ T cells that are specific for lymphocytic choriomeningitis virus (LCMV), 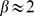 day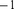 [26] and the doubling time is 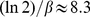 hours. What is the average inter-division time of individual cells? A possible answer is that average inter-division time is simply 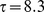 hours, but this implicitly assumes that cells in the population have identical inter-division times (i.e., all cells take exactly the same time to divide). By contrast, if the distribution of inter-division times is exponential (i.e., cells have some chance of dividing right after their previous division), their average inter-division time is 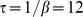 hours which is substantially longer than in the previous case. In general, that the actual average inter-division time of cells cannot be deduced the average population expansion rate 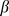 alone. Conversely, a number of different distributions of inter-division times can lead to identical rates of expansions of cell populations. In particular, in the Supporting Information S1 we show that for a population of cells with gamma distributed inter-division times, the total number of cells increases exponentially with the rate 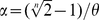 and therefore a number of gamma functions with different scale parameters (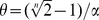) and shape parameters (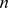 can lead to the identical rate of population growth. This example emphasizes the necessity of a modeling description that allows for an arbitrary distribution of inter-division times [15].

We now review the basic mathematical concepts describing a population of stochastically dividing and dying cells [25], [41], [43]. Immediately after a division, the age, 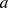, of both daughter cells is zero. We denote the probability of a cell that has divided 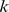 times, to divide for the 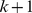-th time in the infinitesimal age interval 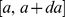 as 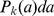; 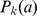 denotes the probability distribution of *inter-division* times. Therefore the probability for a cell to be quiescent up to an age 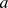 after the 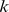-th division, without dividing again, is 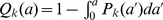. Thus, 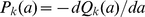. On the other hand, the probability to have the 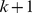-th division in the age interval 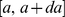 after the 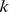-th division is (by definition) the probability of *not* dividing by age 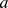, 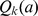, multiplied by the rate of division in the time interval 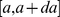, 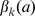, multiplied by the interval length 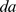, so that 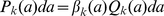. This defines an average *rate* at which cells of age 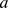 undergo the 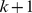-th division: 
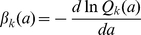
, so that 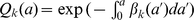. It is important to distinguish between the average division rate 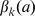 and the inter-division time distribution 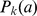; also note that unlike 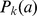, which is a probability density, the quiescence probability 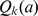 is a cumulative probability distribution and hence is not normalized.

Similar mathematics describes cell death. Instead of dividing, a cell can die at an age 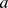, that is in the interval 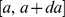 after the previous division, with probability 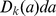. Accordingly, the probability to survive without dying up to an age 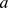 is 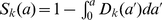. However, the probability of dying in the age interval 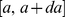 is the product of surviving to age 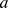, 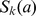 multiplied by the rate of death in the interval 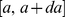, 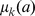, multiplied by the length of the interval 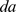, so that 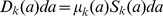. Thus, the average rate of death of cells of age 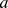 that have divided 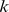 times is 
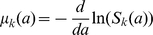
. Also, 
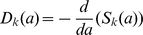
 and 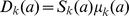. For the case of a simple birth and death process, the birth and death rates are constant and independent of the number of divisions a cell has undergone, 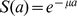, 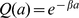 and thus 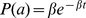, 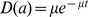, that is the distributions of inter-division and death times are exponential [25], [40], [43]. Further, the probability of a cell living to age 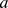 without either dying or dividing is simply 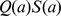.

We denote the number of cells that have undergone exactly 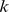 divisions and whose age is in the interval 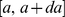 at time 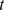 as 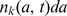. Then, the total number of cells at time 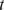 that have undergone exactly 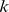 divisions is 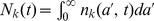 - these cells are termed as belonging to the 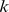-th division class. Cells leave the 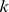-th division class, by either dividing or dying, with the combined rate 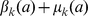, which is written mathematically as [20], [40], [42]

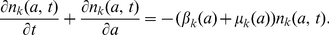
(1)


The cells of age zero in division class 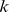 are born from cells in division class 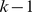. This provides the boundary condition [40]

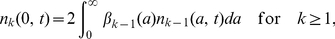
(2)where we have assumed that at each division a cell produces exactly two off-spring.

For simplicity, in the following we assume that the cell population starts at time 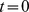 with one cell of age zero, which gives the initial condition for equation (1) 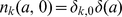, where 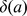 and 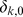 are the continuous and discrete 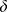-functions, respectively.

Equation (1) can be solved using the method of characteristics [42], [44] (cf. Supporting Information S1) giving for 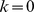


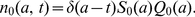
(3)Therefore, the total number of cells in division class zero at time 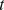 is
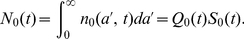
(4)As expected, the total number of cells in the 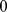-th division class at time 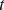, 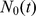, is simply the number of cells that have not divided or died by that time.

Iteratively, one gets for the number of cells of age 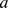 that have undergone one division by time 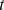, 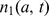:
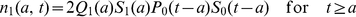
(5)and 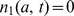 for 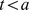. Integrating over age 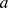 yields the total number of cell in division class 1
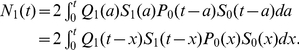
(6)


Equation (6) has a very simple interpretation: namely that the number of cells that have divided once by time 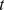 is the number of the descendants of the cells that had their first division at some time 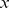, (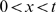), 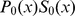, and have remained in the same division class (that is have not divided or died) until the time 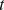, 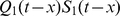.

In general, we get
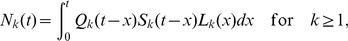
(7)where 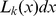 is the number of cells that had their 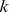-th division in the time interval 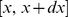, i.e.,
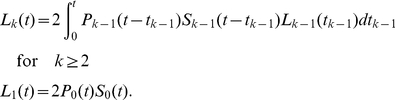
(8)Therefore, by recursion
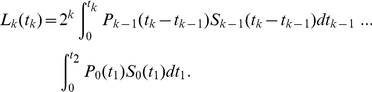
(9)


Equations (7), (8) and (9) are the main results of this section and have the same simple interpretation as the equation for 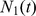: at time 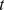, the number of cells in division class 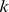 is the number of the descendants of the cells that have had 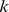 divisions at times 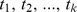 and have survived afterwards, without dividing, until time 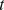.

Also note that the equations (7), (8) and (9) can be compactly written in terms of the Laplace transform:
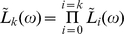


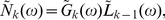
(10)where 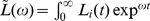 is the Laplace transform of 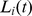 and 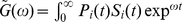 is the Laplace transform of the product 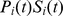.

#### Time-dependent birth and death rates

In principle, the birth and death rates can depend not only on the age of a cell, and the number of divisions it has undergone, but also explicitly on time. Such a situation can arise, for instance, when external signals that influence the birth and death rates change with time.

The age-structured model formulated above can be extended to this case by introducing birth and death rates that depend not only on the age of a cell but also explicitly on time: 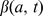 and 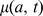, so that the equation (1) becomes
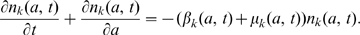
(11)


The survival and the quiescence probabilities 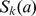 and 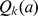 of a cell now explicitly depend on 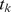, the time of the last division: 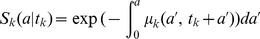 and 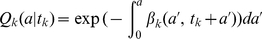, as can be directly derived from the equation (11) using the method of characteristics [42], [44]. Then, similar to equation (9), the number of cells that undergo the 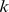-th division at time 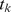, is
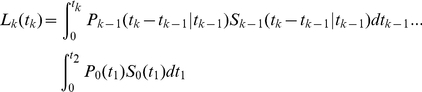
(12)and the total number of cells at time 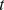 is, similarly to equation (7)
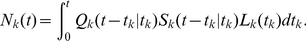
(13)


Although more complicated, the equations of this section have the same simple probabilistic interpretation as equations (7) and (9). Also note that this formulation allows to express any general dependence of the birth an death rates on time.

#### Correlations of the birth and death times between subsequent generations

It has been recently shown *in vitro* that the division and death times of lymphocytes (specifically, B cells) are correlated between the mother and the daughter cells under certain conditions [45]. Whether it is true in general is not known and the underlying causes of such correlations are not fully understood. (for instance, bigger cells might give rise to bigger daughters whose replication times are longer). That is, the parameters of the birth and death time distributions of the daughter cells are not constant but depend on the birth/death times of the previous division. However, a reasonable assumption is that the functional form (shape) of the distribution is the same in all division classes, as it is determined by the same intracellular processes. One way to formalize this mathematically is to introduce the explicit dependence of the distribution parameters in the division class 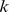 on the actual previous division time in a given cell lineage, 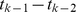. Collectively denoting the parameters of the distribution as 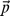, one can the write the general form of the distribution of the inter-division as 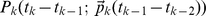. The dependence of the parameters on the previous division time 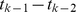 can be arbitrary. For instance, if the mean division time in the division class 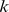 is linearly proportional to the mean division time of the mother, so that 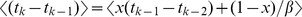 (where 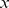 is a numerical parameter), then for gamma-distributed division times, the distribution of times between division 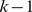 and 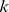 is
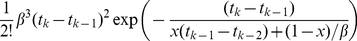
(14)The strength of the correlation can be tuned by varying the parameter 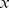.

#### Recruitment rate

**I**n the case of lymphocyte proliferation, the probability distribution of the time to the first division after encounter with an antigen is often different from the subsequent division times. This is due to the different mechanisms involved in initial lymphocyte activation compared to subsequent proliferation of activated lymphocytes [15], [21], [23], [24].

One can model the recruitment of cells into division by simply incorporating it into 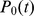, the probability distribution of the time to the first division. However, the time to first division in vivo is determined by two independent processes: the time to the initial encounter with an antigen-presenting cell having enough peptide-MHC on its surface to stimulate the T cell, which defines the recruitment rate, and the time to the first division after that encounter. One can take into account the recruitment rate explicitly denoting the number of unrecruited cells as 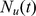. Then 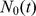 is the number of cells that have been activated by encounter with antigen-presenting cells, but have not divided yet. Accordingly, the probability of not being recruited by time 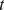 is 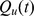 and the probability of not dying by time 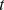 is 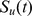. One has to remember that unlike cell division, which is a discrete event, the process of activation by an antigen-presenting cell can be long. Therefore, we define the transition to class 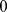 from class 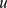 (unrecruited) as a point in the cellular differentiation pathway.

In this case 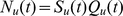, and 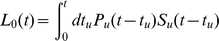. Recalling that the recruitment does not involve a change in cell numbers, the results of the previous section still apply, with an appropriate re-definition of 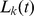:
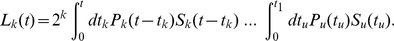



### Branching Processes Theory

Previous authors [15], [36], [38], [39] have used the theory of branching processes to describe proliferation and death of lymphocyte populations. One advantage of using branching process theory is that it allows one to calculate not only the mean number of cells in different division classes, but also the probability of a given number of cells in a given division class. Comparison of the theoretical predictions with the observed statistical variance can help tease apart different mechanisms behind the variability in population behavior. Such analysis lies outside the scope of the present work.

Below we show how the results obtained above using the theory of age-structured populations [25], [36], [41],[43] can be obtained using branching processes.

#### Generation functions for the numbers of cells in different division classes

Given that initially only one cell is present, let us denote the probability that the population contains exactly 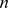 cells in division class 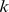 at time 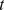 as 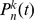. Note that the number of cells in the division class 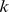 cannot be more than 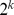. It is convenient to define a generating function for this probability as 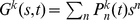 [25], . After the generating function has been computed, one can obtain the probabilities and the mean numbers of cells in different division classes by differentiating 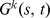 with respect to 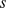. For example, the mean number of cells in a division class 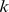 is 
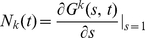
, while 
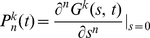
. We now derive expressions for the generation functions 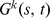 using the methods of the theory of branching processes [25], [41], [43].

We start with the probability that at time 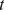 there are no cells in a division class 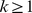, denoted as 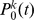. It is a sum of the probabilities that the initial cell has not divided at all by time 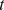, or that it died without dividing, or that it divided once at some time 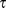 (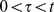), but by time 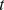 both resulting lineages contain zero cells that divided 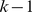 times after the first division. Mathematically,
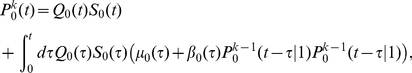
(15)where 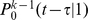 denotes the probability that the progeny of a cell that has undergone the first division at time 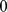 contains 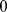 cells that have undergone 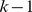 additional divisions by time 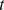.

**Figure 1:**
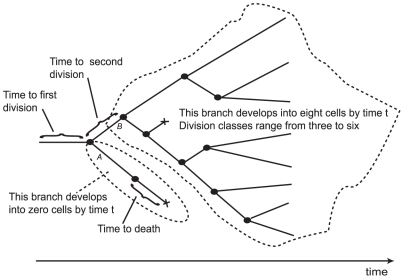
Schematic illustration of the dynamics of a population of proliferating and dying cells, as a branching process. Each node represents a division, and each branch represents a cell lifetime. See text.

Similarly, the probability that at time 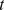 there are 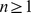 cells in a division class 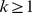, 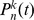, is the probability that the cell has divided at some time 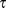, 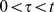, and that the sum of the cell numbers in the division class 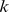 in both resulting lineages is 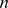 at time 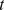. This is expressed mathematically as
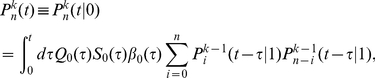
(16)where 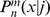 is the probability that one lineage of a cell that divided the 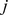-th time at time 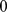 will contain 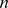 cells that have divided 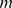 more times by time 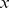 (cf. Figure 1). The 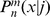 can be calculated iteratively (for 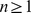):
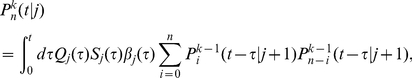
(17)and so on. It is convenient to define division-dependent generating functions 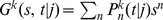. From the above equations, we get an iterative equation for the generating functions
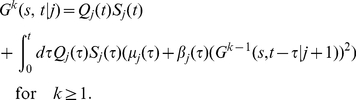
(18)Finally, in the division class zero, 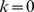, (undivided cells) there can be either one cell or none at all, and the corresponding probabilities are given by
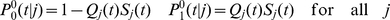
(19)and therefore
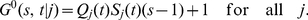
(20)where we have used a generalized notation 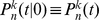 and 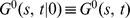


Differentiating the generating function 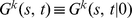 with respect to 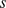, gives the mean numbers of cells in a division class 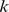, 
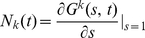
. For instance,
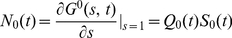
(21)and
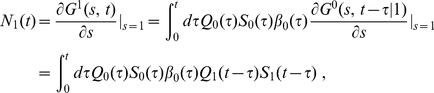
(22)which are identical to those obtained in the previous section using the theory of age-structured populations. This process can be repeated iteratively, resulting in expressions for 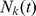
's which are equivalent to those obtained using the theory of age-structured populations, for all 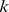 (See Supporting Information S1).

### Comparison of the Existing Theories

In this section we compare models of cell proliferation and death that previously have been used in the context of analysis of lymphocyte dynamics. We show how the existing models can be derived as special cases of the model given in Eqns. (7)–(9).

#### Exponential distribution of inter-division times: linear differential equations

We first show that when the inter-division times and the death times are distributed exponentially (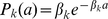, 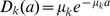 and 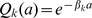, 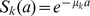), our model reduces to a system of linear differential equations, used by several authors in early studies of lymphocyte proliferation (e.g. [24], [30]). In this case, equations (7),(8) and (9) become
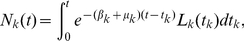
(23)

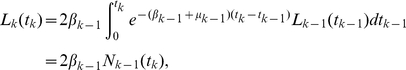
and therefore
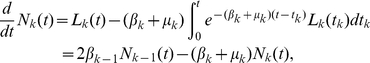
(24)which are identical to the equations used in [24], [30]. Thus, describing the population dynamics of proliferating and dying lymphocytes using linear differential equations of this type [25], [43], is equivalent to the assumption of an exponential distribution of inter-division and death times [24], [25], [43].

#### Smith-Martin-like model

In the framework of this paper (see also Introduction), the Smith-Martin model [18], [20], [24], [32] can be described by the following distribution of inter-division times 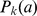, and the probability of not dividing up to age 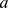, 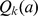, for cells in the division class 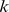:
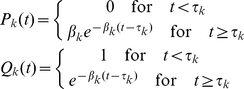
(25)The death times are assumed to be exponentially distributed, so that 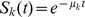.

Using equation (7) we get, 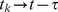


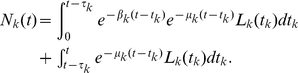
(26)


It should be noted that the classical Smith-Martin model is not exactly identical to that defined by the equations (25): in the classical Smith-Martin model the cells divide upon the exit from the deterministic 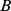 phase and then enter into the stochastic 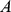 state. In our case the phases are reversed, with the fixed length 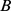 phase occurring first and upon division the cell exits the stochastic 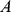 state. Nevertheless, the overall inter-division time distribution as defined in equations (25) is identical to that of the original Smith-Martin model.

The second term in equation (26) describes cells that have divided not longer than 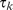 ago, and thus corresponds to the deterministic 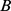 phase of the Smith-Martin model. Similarly, the first term is the number of cells in the exponentially distributed stochastic phase of the cell cycle, i.e., the 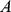 state of the Smith-Martin model. Accordingly, we denote 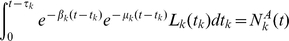 and 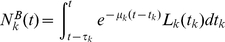.

From equation (8) we get
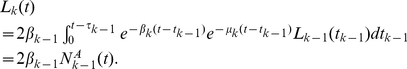
(27)


Consequently,
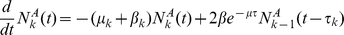
and
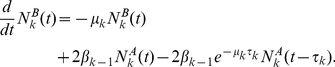
(28)


As mentioned above these equations are similar to those describing the evolution of the Smith-Martin model [19], [20], [24], [34] except for the factor 2 in the equation for 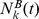. This difference is due to the aforementioned difference between the original Smith-Martin model, and the one defined here by equations (25), where the order of the 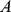-state and 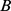-phase is reversed.

#### Cyton and related formulations

Another general model of cell population kinetics based on probability theory is the cyton model [15], [23]. Like our model, it allows one to incorporate arbitrary distributions of the inter-division and death times. It has been recently applied to analysis of the in vitro dynamics of T and B lymphocytes [18], [23]. The connection of the cyton model to the theory of branching processes has been also presented previously [15], [36].

The basic assumption of the cyton model is that the intracellular processes that lead to birth or death of a cell are independent. In mathematical terms, the probability to survive without dividing up to time 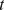 is the product of the corresponding quiescence and survival probabilities: 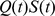, or in other words, the birth and death rates are additive as in equation (1). In this section we show that the formulation obtained on the basis of age-structured population theory is mathematically equivalent to the cyton model.

We start with the expression for number of cells in the 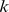-th division class at time 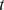, 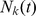, derived in equation (7). After some variable changes, and using the facts that 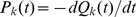, 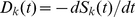, 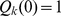 and 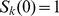, we get
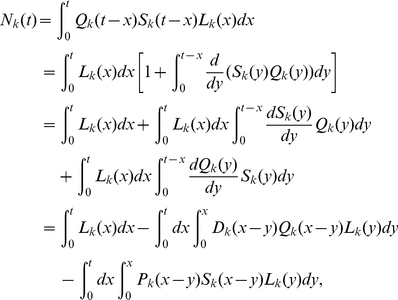
(29)where 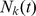 and 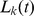 are defined in equations (7),(8),(9).

Equation (29) is identical to the expressions derived for the cyton model [18], [23]. Note that in the present formulation, the “recruitment fraction” of Ref. [23] can be incorporated into the distribution of death times 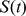. Equation (29) also has a simple probabilistic interpretation: at time 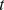, the number of cells in division class 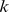 is the number of cells that had their 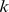-th division at any time 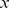 before 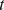 (first term), minus the number of cells that have died (second term), or divided (third term), between time 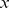 and time 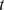 [15], [18], [23].

We also note that for exponentially distributed death times with a uniform death rate 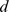 that does not change with division class (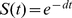 for all 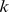), the formulation of this paper reduces to that of Ref. [37].

### Applications to Experimental and Simulated Data

In this section, we explore the computational feasibility of the approach developed in this paper for estimation of parameters of lymphocyte proliferation and death and compare the estimates obtained using different models.

When dealing with experimental data, several important questions arise. First, what distribution of inter-division times to choose? Second, how do estimates of the parameters of the cell division and death depend on the chosen distribution of inter-division times? More generally, are different models distinguishable from the data - can one distinguish between different distributions or, given the distribution, to what extent can one distinguish between different parameter combinations? A general answer to these questions is a complex problem in mathematical statistics, and will not be discussed here. In this paper, we investigate these questions as a ‘case study’, pertinent to analysis of the models of lymphocyte dynamics, putting the theoretical study of the preceding sections in a practical context.

Several of the approaches that are currently used for the analysis of the lymphocyte proliferation and death rely on the use of either simple models with analytical solutions (e.g., ODE models) or numerical solutions or simulations of more complex models (e.g., the Smith-Martin or cyton model). By contrast, we obtain analytical solutions for the number of cells that have undergone a given number of divisions for different distributions of inter-division times, such as the gamma distribution. Once the analytical solution has been obtained, there is no need of further numerical solutions or simulations in order to analyze each particular set of experimental data. This results in large savings of computational time and higher precision of the parameter estimates.

To test the practical feasibility of such an approach and to study the model identifiability issues mentioned above, we first obtained explicit analytical expressions for the model given in eqns. (7)–(9) assuming that the distribution of inter-division times is given by a gamma distribution with a fixed shape parameter (2 or 3) accounting for the possibility that the distributions of inter-division times can be different for undivided and divided cells. The distribution of cell death times was exponential. Explicit analytical expressions for 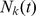 were generated using Mathematica 5.2 (the code is available from the authors upon request). As an example, for the gamma distribution with shape parameter 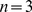 (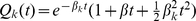 and 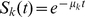 with 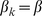 for 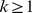 and 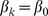 for 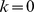), the expressions for the numbers of cells in different division classes are:
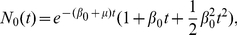
(30)

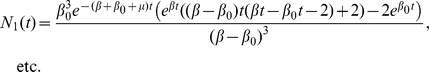



#### Simulated datasets

Briefly, in the simulations the lifetimes of the cells are distributed with a cumulative distribution 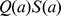, where 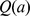 and 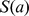 have been defined in the previous sections. At the end of each lifetime, a cell can either divide, with a probability 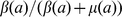 or die, with a probability 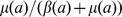. This process exactly implements the division and death process described above. The simulation algorithm was tested for consistency with the mathematical model in the case when both the simulated data and the analytical model were generated using the 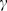-distribution with shape parameter 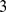 for large initial cell numbers, and excellent agreement between the simulation and the analytical expression was found (data not shown).

**Figure 2:**
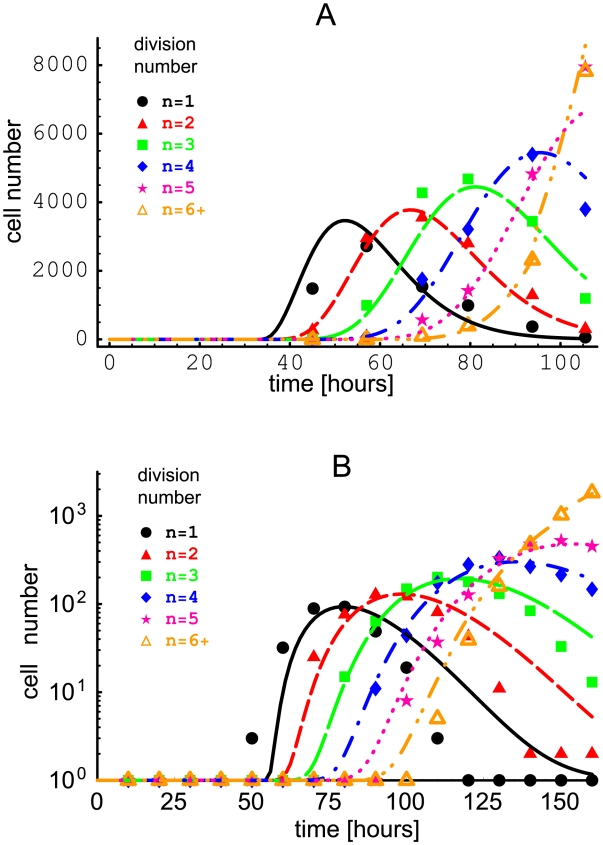
Fitting the solution of the general model to simulated and experimental data. This figure shows the time dependence of the numbers of cells that have undergone 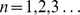 divisions with time for both the data (points) and the model predictions (lines). We use the analytical solution of the model in which the distribution of inter-division times is given by a gamma distribution with the shape parameter 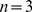 and the death distributed exponentially (cf. text). In panel **A**, the data is the result of simulations of the population starting with 100 cells; inter-division probability distribution is log-normal (see Figure 3). The death rate is exponentially distributed with the mean death rate 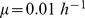. In Panel **B**, the data comes from the experiments with CD4 T cells stimulated in vitro with anti-CD3 antibodies in the presence of 50 U/ml of IL-2 [21]. Parameters providing the best fit of the model to data are given in Table 1. Cells that have undergone 6 or more divisions were lumped into a “6+” division class. Similarly to the previous approaches, we do not fit the dynamics of undivided cells [19], [32]. Standard deviation of the estimated error in the data is 22 (cells) (panel **A**) and 478(cells) (panel **B**).

**Figure 3:**
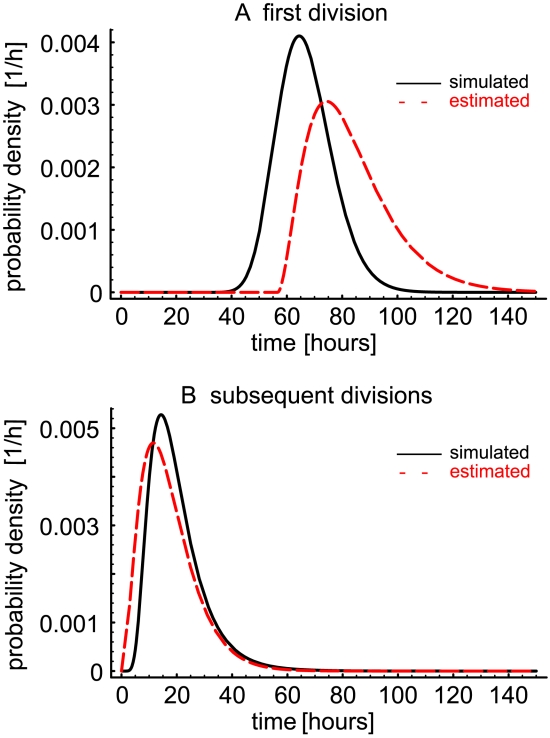
Sensitivity to the choice of inter-division time probability distribution. The dashed lines show the log-normal distribution with the shape parameter 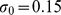 for undivided cells and the scale parameter 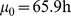 (panel **A**), and 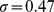 and 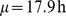 (panel **B**) for divided cells, used in the simulations (see Fig. 2**A**). The solid lines show the inferred gamma-distribution providing the best fit of the simulated data (see text and Table 1).

Subsequently, we tested how reliably the parameters can be estimated from the data when the inter-division time distribution is unknown, which is an important question because this is typically the experimental situation. To this end, from simulations we generated several datasets in which cell divisions were distributed in accord with either a log-normal distribution, a gamma-distribution with shape parameter 3, or a Weibull distribution, starting with an initial number of cells 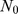. The parameters of the inter-division time distributions were the same in all division classes except for 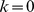, which was different, which is a common situation for lymphocytes stimulated to proliferate in vitro. For small initial cell numbers, simulations result in stochastically variable data (see Fig. 2A). As expected, in the case when the datasets were fitted using the analytical expressions with the same inter-division time distribution as used in generating them (gamma-distribution with shape parameter 3), we were able to obtain a good fit and recover the parameters used to simulate the data (results not shown). More importantly, we also were able to obtain good fits even in the cases when the actual distribution of inter-division times in the simulated data was relatively different from the gamma distribution used for fitting (e.g., when the simulated data generated using a log-normal distribution, was fitted with the analytical solutions for gamma-distribution with shape parameter 3 - cf. Fig. 2A ); both birth and death times could be estimated reliably (see Fig. 2A, Table 1 and Fig. 3). Similar results were obtained when we fitted data generated using Weibull distribution for cell division times with Eqn. (31) (results not shown). The model with gamma distributed interdivision times (and 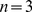) can fit well various data and recover model parameters properly. By contrast, we generally obtained poor fits of simulated (and experimental) data when we used a model with gamma distributed inter-division times with the shape parameter 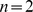 (not shown). These results suggest that model that has a different underlying distribution of inter-division times than the data can still provide reasonable fits of the data, but this need not be the general case.

**Table 1:** Estimates of the parameters providing the best fit to the simulated and experimental data.

	Simulated data		Experimental data	
	Actual	Estimated	Estimated gamma	Estimated SM
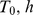	66.7	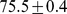	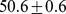	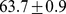
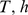	20	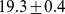	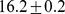	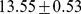
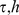	0	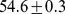	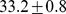	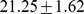
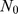 cells	100	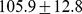	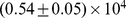	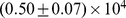
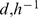	0.01	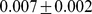	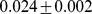	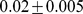

Left column: fit of the simulated dats with the general model of eqns. (7)(described in the text and Figure 3). Right column: comparison of the fits of the experimental data with either the general model of Eqns. (7) or Smith-Martin model. or Smith-Martin model. Here 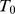 and 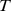 are the average times of the first and the following cell divisions, 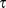 is the delay before any cells undergo their first division, 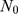 is the initial number of cells, and 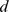 is the death rate of dividing cells. Standard deviations for parameters were calculated by bootstrapping the residuals 100 times [32], [48]. Simulated data was generated using a log-normal distribution of inter-division times as shown in Fig. 3, with the exponential death rate 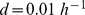.

**Figure 4:**
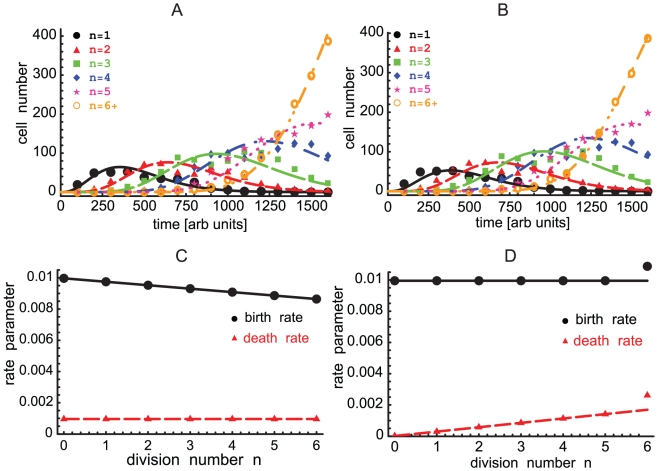
Division-dependent birth and death rates. In simulations, the population starts with 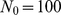 cells whose proliferation times are distributed in accord with gamma distribution with the shape parameter 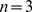 and the rate parameter 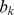 that depends on the number of cell divisions 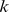, 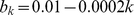. Cells die at the constant rate 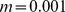. Panels A and C: fit of the simulation data with the model predictions for with division-dependent birth rate (equations not shown). The estimated parameters of the fit are: 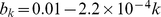, 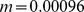, 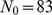. Panel A: fit of the model to the data; Panel C: the estimated dependence of the birth and death rates on the number of cell divisions 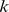. Panels B and D show the fit of the simulated data with the model that assumes division-dependent death rate. The estimated parameters are 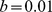, 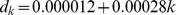, 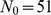. Panel B: fit to the data; Panel D: the estimated changes in the death rates with the number of cell divisions. The quality of the model fit to data is similar in both cases as judged by the mean square deviation; the standard deviation of the fit was 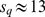 (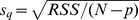 where 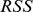 is the residual sum of squares, 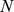 is the number of data points and 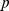 is the number of model parameters).

It has been suggested in several works that the division rate might depend on the division class [19], [23], [32], [38]. Can such dependence be unambiguously determined from the cell division data alone? To this end, we simulated cell dynamics choosing the inter-division times to obey a gamma distribution with shape parameter 3, while the division rate parameter increased linearly with the number of divisions that the cell had undergone (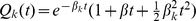 with 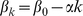, where 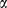 is a constant). The cell death times were chosen to be distributed exponentially. The resulting data were fitted with two different solutions of the general model in which either the birth rate, 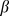, *or* the death rate, 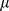, were assumed to vary linearly with the number of cell divisions 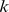. We found that in both cases, the models could fit the data with good quality. The differences between the standard deviation of the fits were not much higher than the expected statistical error due to the stochasticity of the cell division process (see Fig. 4). This result indicates that if CFSE data contain information on division-dependence of parameters, it might be difficult to know which parameters change with division class without additional experimental data. A similar conclusion was also reached in a previous study [32].

**Figure 5:**
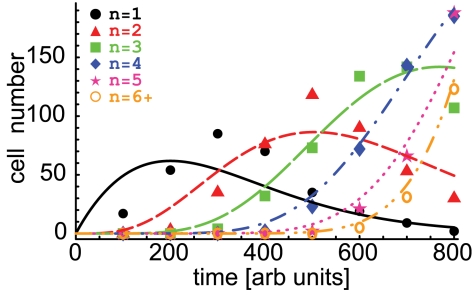
Time-dependent division rates. Here, we simulate the dynamics of cells assuming that their division times are distributed in accord with exponential distribution and the division rate changes with time. The data were fitted with the model in which birth rate changes linearly with the number of cell divisions. Interestingly, the model describes the data reasonably well, at least for the later divisions, suggesting that changes in rates of cell division over time may look similar as that over the number of cell divisions, and that additional experimental data is needed to discriminate between time - and division-dependence.

A related important question arising in the context of analysis of the cell division data (in the case of variable inter-division times) is whether the division rates are linked to the division class, or to the time since stimulation. Distinguishing between these two possibilities can provide important insights into inter- or intra-cellular mechanisms of regulation of the lymphocyte number during immune response. To provide insight into this aspect of data analysis, we have simulated population expansion with exponentially distributed inter-division times where the birth rates increase linearly with time and fitted the simulated data with the predictions of the model where the birth rates increase with the division class. Interestingly, the model can fit the data reasonably well, at least for the later divisions. However, the recovered parameters were not close to the actual ones, suggesting that although the populations where the rates of cell division change over time may look similar as those where the rates change with the number of cell divisions, additional experimental data is probably needed to discriminate between time- and division-dependence. These results are summarized in Fig. 5.

**Figure 6:**
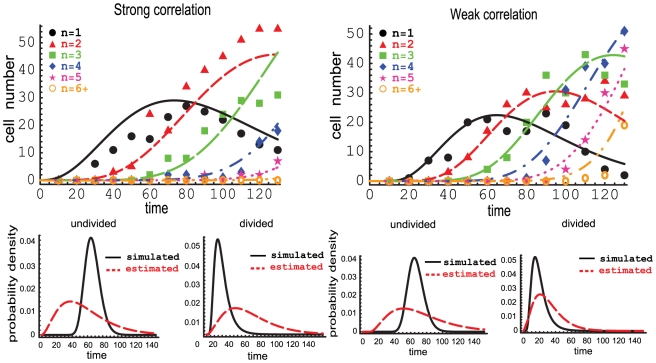
Division times of daughter cells correlated with that of the mother. We simulate the dynamics of a cell population with gamma distributed inter-division times assuming strong or weak correlation between average division times of mothers and daughters (see Section). In the case of the weak correlation (*left panels*), the data can be fit with the uncorrelated model reasonably well, although the recovered parameters are different from the parameters used in the simulations. For strong correlations, the model is not able to describe the simulated data (*right panels*).

Finally, we explored whether the correlations in division times between daughter and mother cells can be inferred from the division data alone. To this end, we generated simulated datasets where the division times of the daughter cells are distributed according to the gamma-distribution with shape parameter 3 and are either weakly or strongly correlated with the division times of the mother cells (see text above equation (14)). The resulting data have been fitted with the model with *uncorrelated* division times obeying the same gamma-distribution with shape parameter 3. In the case of weak correlations, the model could describe the data reasonably well, while for strong correlations the fit was reasonable only for higher division classes. However, the errors of the fit were high and the parameters for cell division could not be determined correctly ( See Fig. 6). These results indicate that in some cases, even if data have intrinsic correlations between generation times of mothers and daughters, model without such correlations can describe the data well. Furthermore, it might be difficult to obtain unambiguous inference based on the cell division data alone.

#### Experimental data

Next, we fitted the model solutions to data obtained in experiments in which CD4+ T lymphocytes were stimulated with anti-CD3 antibodies *in vitro* in the presence of a high concentration of exogenous interleukin-2 (IL-2) [21]. These data have been fitted before with the Deenick et al. model [19] and the Smith-Martin model [32]. The formulation developed in this paper fits these data as well as both previous models. The estimated death rate of dividing cells obtained from the fit of the model to the data obtained using the gamma-distributed inter-division times was almost identical to those obtained with the Smith-Martin model [32]. This is not very surprising since all of these models assume that death is exponentially distributed. However, the average time of the first division and the average inter-division time of divided cells obtained from our fit are somewhat different from that obtained using the Smith-Martin model (cf. Fig. 2B and Table 1). This and the above result indicate that estimates of important kinetic parameters, such as the average inter-division time, may depend on the model used to fit the data (see [19], [32]).

To summarize, we have shown that the analytical solutions of the general model obtained in this paper can fit artificial and experimental data with reasonable quality providing a parameter estimation tool complementary to existing models.

## Discussion

Understanding the mechanisms of the immune response requires, among other things, quantitative measurements of the kinetics of lymphocyte proliferation and death. Recently, several different mathematical descriptions of the kinetics of lymphocyte proliferation and death have been used in order to estimate birth and death parameters of lymphocyte populations during an immune response [15], [17]–[21], [23], [24], [26], [27], [30], [32], [34]–[36], [42], [46]. These works lay a foundation for the quantitative analysis of immune response kinetics.

In order to compare the estimates obtained using different models and make meaningful inference, it is important to understand the differences and similarities in the mathematical structures of different models. It is also important to understand how the estimates obtained using different models are sensitive to the choice of model characteristics, such as the shape of the inter-division time distribution.

In this paper we have provided a mathematical comparison of a general model based on the theory of age- and generation-structured populations with other formulations (such as Smith-Martin, cyton and branching processes) and show under what conditions they are mathematically equivalent. Based on the mathematical formulation, we developed an algorithmically and conceptually simple way for estimation of kinetic parameters of lymphocyte proliferation and death. The algorithm was used for analysis of simulated and experimental data.

It is important to emphasize that in the majority of situations, the true distribution of division and death times of proliferating cells is unknown and estimating the rates of cell division and death could strongly depend on the model used to fit the data. We have shown that in some cases, different models can fit equally well particular types of data while in other cases different models lead to different parameter estimates. Our novel approach and analytical solutions of the model with gamma distributed inter-division times adds to the arsenal of models currently available to experimentalists. This and other models can therefore be used to test whether the estimates for the rates of cell division and death in a particular experimental situation depend on the model used to fit the data. In those cases when different models yield similar estimates for the rates of cell division and death (e.g., average inter-division time, the probability of death per division, etc.) one can be confident that these parameters are estimated robustly. In cases when different models yield different parameter estimates, additional information is needed to rule out alternative models for cell division (see also [47]).

Lastly, although our work was motivated by problems in lymphocyte population kinetics, the methods are applicable more broadly. For example, the spread of a viral infection could be modeled by a simple generalization of the type of branching process used here, where each infected cell rather than having exactly two offspring, gives rise to a number of new infected cells in the next generation.

## Materials and Methods

The analytical calculations were performed with pencil and paper with the help of Mathematica 6 package. The simulations were written on C and compiled and executed under UNIX or Windows operating systems. Data fitting was performed in Mathematica 5.2.

## Supporting Information

Supporting Information S1 (0.08 MB PDF)
