# Supplementary material for: Stochastic Models of Lymphocyte Proliferation and Death
Source: PLoS One. 2010 Sep 30;5(9):e12775. doi: 10.1371/journal.pone.0012775 (PMC2948000; doi:10.1371/journal.pone.0012775)
Supplement: Supporting Information S1 — (0.08 MB PDF) [file pone.0012775.s001.pdf]

Appendix

Derivation of the equations of Section 1 in the main text

Using the method of characteristics [1,2] we get from equations (1) and (2) in the main text

$$n_0(a,t) = n_0(0,t-a)e^{-\int_0^a \beta_0(a')da'}e^{-\int_0^a \mu_0(a')da'} \quad \text{for} \quad a < t \tag{1}$$
$$n_0(a,t) = n_0(a-t,0)e^{-\int_{a-t}^a \beta_0(a')da'}e^{-\int_0^a \mu_0(a')da'} \quad \text{for} \quad a \geq t \tag{2}$$

where $\Theta(x)$ is the step function: $\Theta(x) = 1$ for $x \geq 0$ $\Theta(x) = 0$ for $x \leq 0$. Using the initial condition $n_0(a,0) = \delta(a)$ one gets

$$n_0(a,t) = \delta(a-t)S_0(a)Q_0(t) \tag{3}$$

Therefore, the total number of cells in division class zero at time t is

$$N_0(t) = \int_0^\infty n_0(a',t)da' = Q_0(t)S_0(t) \tag{4}$$

As expected, the total number of cells in the 0 division class at time t, $N_0(t)$, is simply the number of cells that have not divided or died by that time.

Iteratively, one gets for the number of cells of age a that have undergone one division by time t, $n_1(a,t)$

$$n_1(a,t) = Q_1(a)S_1(a)n_1(0,t-a) = 2Q_1(a)S_1(a)\int_0^\infty \beta_0(a')n_0(a',t-a)da' \quad \text{for} \quad t \geq a \tag{5}$$

and zero for $t < a$, where we have made use of equation (2). Using equation (3) and the fact that $P_k(t) = \beta_k(t)Q_k(t)$, we get

$$n_0(a,t) = \delta(a-t)S_0(a)Q_0(a) \tag{6}$$

Therefore, the total number of cells in division class zero at time t is

$$N_0(t) = \int_0^\infty n_0(a',t)da' = Q_0(t)S_0(t) \tag{7}$$

As expected, the total number of cells in the 0 division class at time t, $N_0(t)$, is simply the number of cells that have not divided or died by that time.

Iteratively, one gets for the number of cells of age a that have undergone one division by time t, $n_1(a,t)$

$$n_1(a,t) = Q_1(a)S_1(a)n_1(0,t-a) = 2Q_1(a)S_1(a)\int_0^\infty \beta_0(a')n_0(a',t-a)da' \quad \text{for} \quad t \geq a \tag{8}$$

and zero for $t < a$, where we have made use of equation (2). Using equation (3) and the fact that $P_k(t) = \beta_k(t)Q_k(t)$, we get

$$n_1(a,t) = 2Q_1(a)S_1(a)P_0(t-a)S_0(t-a) \quad \text{for} \quad t \geq a \tag{9}$$

and $n_1(a,t) = 0$ for $t < a$. Finally, integrating over the age a

$$N_1(t) = 2\int_0^t Q_1(a)S_1(a)P_0(t-a)S_0(t-a)da = 2\int_0^t Q_1(t-x)S_1(t-x)P_0(x)S_0(x)dx \tag{10}$$

Calculation of the $N_k(t)'s$ from the branching process theory

The subsequent mean numbers of cell can be calculated in the same manner. Using the fact that $G(1,t|j) = 1$ for all j, from Eqns.(15)-(20) in the main text one gets

$$N_2(t) = \frac{\partial G^2(s,t)}{\partial s^2}|_{s=1} = \int_0^t d\tau Q_0(\tau)S_0(\tau)\beta_2(\tau)2\frac{\partial G^1(s,t-\tau|1)}{\partial s}|_{s=1} \tag{11}$$

$$2\int_0^t d\tau Q_0(\tau)S_0(\tau)\beta_0(\tau)\int_0^{t-\tau} dx Q_1(x)S_1(x)\beta_1(x)2\frac{\partial G^0(s,t-\tau-x|2)}{\partial s}|_{s=1} = \tag{12}$$

$$\int_0^t P_0(\tau)S_0(\tau)\int_0^{t-\tau} dx P_1(x)S_1(x)Q_2(t-\tau-x)S_2(t-\tau-x) \tag{13}$$

Making a substitution $\tau - x = t_2$ and $\tau = t_1$ we get

$$N_2(t) = 2^2\int_0^t dt_2\int_)^{t_2} dt_1 P_0(t_1)S_0(t_1)P_1(t_2-t_1)S_1(t_2-t_1)Q_2(t-t_2)S_2(t-t_2) \tag{14}$$

which is identical to the Equation (7) in the main text for. This process can be continued iteratively.

Approximate population expansion rate

In this section we derive an approximate expression for the population expansion rate in the case with no cell death and the distributions of the division and death times do not change from division to division, that is $Q_k(t) = Q(t)$ and $S_k(t) = S(t)$ for all k.

From equations (7),(8) in the main text we get

$$\begin{aligned} N_k(t) &= \int_0^t Q(t-t_k)S(t-t_k)L_k(t_k)dt_k = \\ &= \int_0^t Q(t-t_k)S(t-t_k)dt_k\int_0^{t_k} P(t_k-t_{k-1})S(t_k-t_{k-1})L_{k-1}(t_{k-1})dt_{k-1} \end{aligned} \tag{15}$$

Making the substitution of variables $x = t-(t_k - t_{k-1})$ we get

$$\begin{aligned} N_k(t) &= 2\int_0^t P(t-x)S(t-x)dx\int_0^x Q(x-t_{k-1})S(x-t_{k-1})L_{k-1}(t_{k-1})dt_{k-1} \\ &= 2\int_0^t P(t-x)S(t-x)N_{k-1}(x)dx \quad \text{for} \quad k = 1,2,... \end{aligned} \tag{16}$$

Summing equations (16), using Neumann series [8] and adding equation (4) for $N_0(t)$, produces an elegant equation for the total number of cells at time t, $N(t) = \sum_{k=0}^{\infty} N_k(t)$

$$N(t) = Q(t)S(t) + 2\int_0^t P(t')S(t')N(t-t')dt' \tag{17}$$

This equation is known as the 'renewal equation' and arises in the context of the theory of renewal and branching processes [3–6]. This equation has a simple probabilistic interpretation: the number of cells at time t is the sum of all the progeny of the cell that has divided at some time during the interval $[0,t]$. A deeper connection to the theory of branching processes is provided in the main text and in [7].

Let us look for an asymptotic (i.e., large time t) solution for the integral equation (17), which determines the populations size $N(t)$, in the form $N(t) = e^{\alpha t}$ [5]. Equation (17), with $S(t) = 1$ as in the examples, then becomes

$$N(t) = Q(t) + 2\int_0^t P(t-t')N(t')dt' = Q(t) + 2\int_0^t P(t')N(t-t')dt' \tag{18}$$

Thus, with $N(t) = e^{\alpha t}$

$$e^{\alpha t} = Q(t) + 2\int_0^t P(t')e^{\alpha(t-t')}dt' \tag{19}$$

Dividing both sides by $e^{\alpha t}$, it reduces to

$$1 = Q(t)e^{-\alpha t} + 2\int_0^t P(t')e^{-\alpha t'}dt' \tag{20}$$

In the asymptotic limit of large times, $t \gg 1/\alpha$, we get an implicit equation for α

$$\frac{1}{2} = \int_0^\infty P(t)e^{-\alpha t}dt \tag{21}$$

For instance, for $P(t) = \theta^n t^{n-1}/(n-1)!e^{-\theta t}$, after integration it can be solved to yield $\alpha = (\sqrt[n]{2} - 1)/\theta$ and $N(t) \simeq \exp(\alpha t)$ for $\theta t \gg 1$. It is also useful to note that $\int_0^\infty P(t)e^{-\alpha t}dt$ is the Laplace transform of $P(t)$ [5].

References

1. Bernard S, Pujo-Menjouet L, Mackey M (2003) Analysis of cell kinetics using a cell division marker: mathematical modeling of experimental data. Biophys J 84: 3414–3424.

2. Evans L (1998) Partial Differential Equations. American Mathematical Society Publishing.

3. Bharucha-Reid A (1960) Elements of the Theory of Markov Processes and their Applications. McGraw-Hill

4. Bharucha-Reid A (1954) Age-dependent branching stochastic processes in cascade theory. Phys Rev 96:751–753.

5. Harris T (1963) The Theory of Branching Processes. Dover Publications.

6. Kimmel M, Axelrod A (2002) Branching Processes in Biology. Springer-Verlag.

7. Subramanian VG, et al. (2008) Determining the expected variability of immune responses using the cyton model. J Math Biol 56:861-892.

8. Arfken G, Weber H (2001) Mathematical Methods for Physicists, 4th edition, Academic Press.
